# Cloning and expression analysis of *BmYki* gene in silkworm, *Bombyx mori*

**DOI:** 10.1371/journal.pone.0182690

**Published:** 2017-08-09

**Authors:** Wenhui Zeng, Riyuan Wang, Tianyang Zhang, Chunying Gong, Weidong Zuo, Rongpeng Liu, Yao Ou, Hanfu Xu

**Affiliations:** 1 State Key Laboratory of Silkworm Genome Biology, Southwest University, Chongqing, China; 2 College of Biotechnology, Southwest University, Chongqing, China; 3 Institute of Sericulture and Systems Biology, Southwest University, Chongqing, China; University of Dayton, UNITED STATES

## Abstract

The transcriptional coactivator Yorkie(Yki), is a critical downstream effector of the Hippo(Hpo) signaling pathway that controls organ size through the regulation of cell proliferation and apoptosis. During the past ten years the biological function of Yki has been studied extensively in *Drosophila* and a few other insects, however, little is known about it in the silkworm, *Bombyx mori*, a major research model of lepidopteran insect. Here, we describe the isolation, characterization and expression of the *B*. *mori* Yki ortholog, BmYki. The coding sequence of the *BmYki* was 1314 bp in length, encoding a protein of 437 amino acids containing two conserved WW domains. *BmYki* transcripts were ubiquitous but not abundant in all detected tissues and developmental stages. Comparatively, it was expressed at pretty high level in silk glands and at the stage of fifth-instar day-3 larvae. Overexpression of *BmYki* in cultured *B*. *mori* embryonic cells significantly promoted transcription of genes associated with cell proliferation and apoptosis, indicating that *BmYki* functions in the regulation of organ growth-related biological processes. Interestingly, transcription of silk protein-coding genes and transcription factors regulating the synthesis of silk proteins was downregulated remarkably, suggesting that *BmYki* was involved in the regulation of silk protein synthesis. This study provides new insights into the role of *BmYki* in Hpo pathway regulation in silkworm.

## Introduction

Precise control of organ size is a key feature of metazoans and a crucial process during animal development and regeneration[1]. How the animal organs grow to be the right size is one of the central mysteries of biology. Developmental genetics over the past decade have revealed the size of functional organs is a consequence of regulation of cell number and/or cell size, which are generally achieved by coordinatly regulating cell growth, proliferation, and apoptosis[1–2]. Several signaling pathways have been implicated in such regulation, including the Wnt, Hedgehog, Transforming growth factor β(TGFβ), Notch, Wingless, Myc, Target of rapamycin(TOR), Jun N-terminal kinase(JNK), and Hippo(Hpo) pathway[3–10]. Studies on these pathways will provide important entry points for clarifying mechanisms of organ size control.

The Hpo pathway firstly discovered in *Drosophila* and subsequently found in mammals and a few other insects[11–16], is now appreciated as a key regulator of organ growth in flies and mammals. It also plays crucial roles in other biological processes, such as tissue homeostasis and regeneration, cell fate determination, stem cell proliferation, and innate immunity[1,17–18]. It has been defined that core to the Hpo pathway is a kinase cascade composed of tumor suppressors Hpo, Salvador(Sav), Mob-as-tumor-suppressor(Mats), and Warts(Wts)[11–12,19–22], which ultimately phosphorylates and inactivates the transcriptional coactivator Yorkie(Yki), the most critical downstream effector of the Hpo pathway[23–24]. In *Drosophila*, loss of Hpo signaling activated Yki and led to accumulation of it in the nucleus and therefore induced expression of target genes that positively regulate cell growth, survival, and proliferation[25–26]. Overexpression of Yki resulted in organ overgrowth characterized by excessive cell proliferation and diminished apoptosis, and rescued the phenotype of Hpo signaling activation[23,27]. In *Helicoverpa armigera*, knockdown of Yki in the epidermal cell line(HaEpi) induced increased activation of cell apoptosis-related genes, whereas overexpressed Yki in HaEpi cells promoted cell proliferation-related genes[28–29]. Taken together, these evidences hint that Yki is a pivotal "switch" to uncover the role of Hpo pathway in organ size control.

We have previously identified the Yki ortholog(designated as BmYki) from the lepidopteran model insect, the silkworm *Bombyx mori*, yet whether it functions in regulating organ size has not been fully defined. In this study, we isolated the *BmYki* gene from three strains of *B*. *mori*, and further investigated the expression of *BmYki* and its function in the regulation of its downstream targets related to organ growth. Finally, we provided evidences that *BmYki* is involved in the synthesis of silk proteins in *B*. *mori*.

## Materials and methods

### Animals and cell lines

Three *B*. *mori* strains, *Dazao*(diapaused strain), *Nistari*(non-diapaused strain) and *LH*(non-diapaused strain), were obtained from Silkworm Gene Bank of Southwest University and reared with fresh mulberry leaves in the laboratory at 25~27°C. The cultured *B*. *mori* embryo cells(BmE) and ovary-derived cells(BmN) were stored in our laboratory and grown in Grace’s insect medium containing 10% fetal bovine serum at 27°C.

### Gene cloning and sequence analysis

The nucleotide sequence of BmYki(BGIBMGA003638) previously identified from the database of SilkDB(http://www.silkdb.org/silkdb/), was used to design the specific primers(Table 1) and perform real-time polymerase chain reaction(RT-PCR) using the cDNA template of three *B*. *mori* strains, respectively. The PCR products were subcloned into the pEASY-T5 vector(TransGen) and verified by DNA sequencing. Alignment of the sequences was carried out using software ClustalX[30]. Searches of CDS domain and exton/intron were carried out through the use of GENSCAN(http://genes.mit.edu/GENSCAN.html) and SMART(http://smart.embl-heidelberg.de/). The *BmYki* gene isolated from *Dazao* strain was used for subsequent experiments.

**Table 1:** Primer sequences.

	Genes	Forward primer(5'-3')	Reverse primer(5'-3')
**A**	*BmYki*	ggatccATGGCTCTCAACTCGGACGGT	gcggccgcTTACAGCCACGTGAGTACGTTGTC
**B**	*BmYki*	ggatccATGGCTCTCAACTCGGACGGT	gcggccgcCAGCCACGTGAGTACGTTGTC
**C**	*BmYki*	CGAAGAGTACAAGTAATACGACAA	TACGAGCTGCGTGATTAATG
**D**	*Myc*	CTGTATGTGGGCAGGTTCG	GTGGTTCATCGCCGTCTAA
	*Ras1*	TGCGGGAGGCGTTGGTAAA	CAGCCGTGTCCAGGATGTCG
	*E2F1*	TACAGCAGACCGTCCAGTT	CCGCCGCTATGTTCAAAT
	*CyclinE*	GTCCACCCCACACTCTAATAAA	TCAGCCCAAGACAATCCAG
	*Diap1*	GATTGGGAAAGCGATGACG	GCCTCCGACTTCACCTTCTG
	*Diap2*	GAGTGCCGAAACGACAATA	AGTGCCTCTGAAGTCTGGAA
	*Caspase1*	AGGTGATAAGTTAGATGGTGG	TGTGTTTCTCCAAGAGTAATAA
	*Caspase9*	TGCGTGTTCCTGGTGGTGTC	TCGGGAGGTCCGTGAAGTTG
	*Kibra*	CGACCTATCCGAAGACGACTG	GCACGGGTTCTTACATTCCAC
	*Expanded*	AGTTCCGATTGCGTTGATG	ATCTGTTGGGTCACTTCCG
**E**	*fibH*	CAGGGGATACGGACAAGGT	TTCACACAAGGCAGTGCTCT
	*fibL*	GGAGGTGGAAGAATCTATGAC	TGTAGGCAGCGATGTTGT
	*P25*	GGGTCTGCCCATCTTCCAC	CTCGCCAGCCAGTTCCTCT
	*Ser1*	GGTCCAGAAGGCGTGTCGT	ATTGTCCCGCAGAAGCAGAT
	*Ser2*	CCAGGAGGATAACGACAGC	TGGAGAACTTGTCGTGGGT
	*Ser3*	TACAGGTATGGCTGCGGA	TCATCGGAGTCCTCGTCAT
	*Sage*	ATTACGAGCCCAAGAGGAT	CCACTACGGTGTCACGAAC
	*Dimm*	CACCGAATCTCCTGACCAA	CATCACTTCCGCTACCACTAT
	*SGF1*	GCAGCACCCGTTCAGCATC	GCGGCGACTGGTAGTAGTTATCC
	*SGF2*	CCTGATACCTCGCTACTTCCG	CGTGATGCTGGTGTTGTGG
	*SGF3*	GAAACCGTCCGCTCAAGAAA	GTTCGGTGGCGTCATCCTC
**F**	*sw22934*	TTCGTACTGGCTCTTCTCGT	CAAAGTTGATAGCAATTCCCT

A: Primers for gene cloning; B: Primers for subcellular localization construct; C-F: Primers for qRT-PCR analysis. Lowercase letters indicate restriction enzyme sites.

### Construction of expression vectors

*pSL[BmA4-BmYki/EGFP-Ser1pA](abbreviated as BmYki-EGFP)*. The coding sequence of the BmYki with the stop codon deleted was PCR-amplified using specific primers(Table 1), digested with *BamH* I and *Not* I, and inserted into the pSL[BmA4-BN/EGFP-Ser1pA] vector, to generate the vector pSL[BmA4-BmYki/EGFP-Ser1pA]. Expression of the BmYki-EGFP fusion protein was controlled by the promoter of *B*. *mori actin4* gene(BmA4).

*pB[UAS-BmYki*,*3×P3EGFP](abbreviated as UASBmYki)*. The coding sequence of BmYki was digested with *BamH* I and *Not* I, and inserted into the pUC57S[10×UAS-B/N-Ser1pA] vector, then the fragment UAS-BmYki-Ser1pA was subcloned into the *Asc* I site of the pB[3×P3EGFPafm][31], to generate the vector pB[UAS-BmYki,3×P3EGFP].

*pB[BmA4-Gal4*,*3×P3DsRed](abbreviated as A4G4)*. The synthesized Gal4 sequence was digested with *BamH* I and *Not* I, and inserted into the pUC57S[BmA4-B/N-Ser1pA] vector, then the fragment BmA4-Gal4-Ser1pA was subcloned into the *Asc* I site of the pB[3×P3DsRedaf][31], to generate the vector pB[BmA4-Gal4,3×P3DsRed].

### Subcellular localization analysis

A 100 μL mixture containing 3 μg of BmYki-EGFP plasmid DNA was mixed with 100 μL Grace’s insect medium(without antibiotics) and incubated for 20 min at room temperature, then mixed with BmN cells. After 72 h culture at 27°C, cells were harvested, washed three times with 1×PBS, fixed with 4% paraformaldehyde for 10 min, dyed with 0.1% DAPI for 20 min at room temperature, and then washed three times with 1×PBS. The fixed cells were mounted in slides and observed using an FV1000 confocal microscope(Olympus, Japan). Images were processed using Photoshop 5.0.

### Quantitative RT-PCR(qRT-PCR) analysis

To analyze expression patterns of *BmYki*, total RNA from different tissues and developmental stages of *Dazao* strain were isolated and used to prepared cDNA templates using cDNA synthesis Kit Manual(Takara). qRT-PCR was carried out in 20 μL solution, which contains 5 ng of cDNA templates, 10 μL of 1×SYBR@ Green I taq(Takara) and 0.5 mM of each primer. The 7500FAST Real-Time PCR System(ABI, USA) was employed. The eukaryotic translation initiation factor 4A (silkworm microarray probe ID: sw22934) was used as an internal control[32].

To determine the mRNA level of target genes in BmE cells overexpressing *BmYki*, the cells transfected with 3 μg mixture of UASBmYki(400 ng/μL) and A4G4 plasmid DNA(400 ng/μL) were harvested after 72 h culture at 27°C and used to prepare cDNA templates. mRNA levels of each target gene were measured by qRT-PCR as described above. All primers used in qRT-PCR were summarized in Table 1.

## Results and discussion

### Isolation and sequence analysis of BmYki

**Fig 1:**
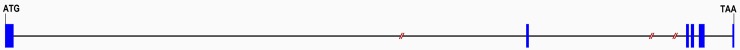
Schematic representation of the gene structure of BmYki. The exons(blue boxes) and introns(black lines) are shown. Double slashes indicate gaps in genomic sequences. The length of exon 1 ~ exon 6 is 473, 143, 141, 158, 303, and 96 bp, respectively.

**Fig 2:**
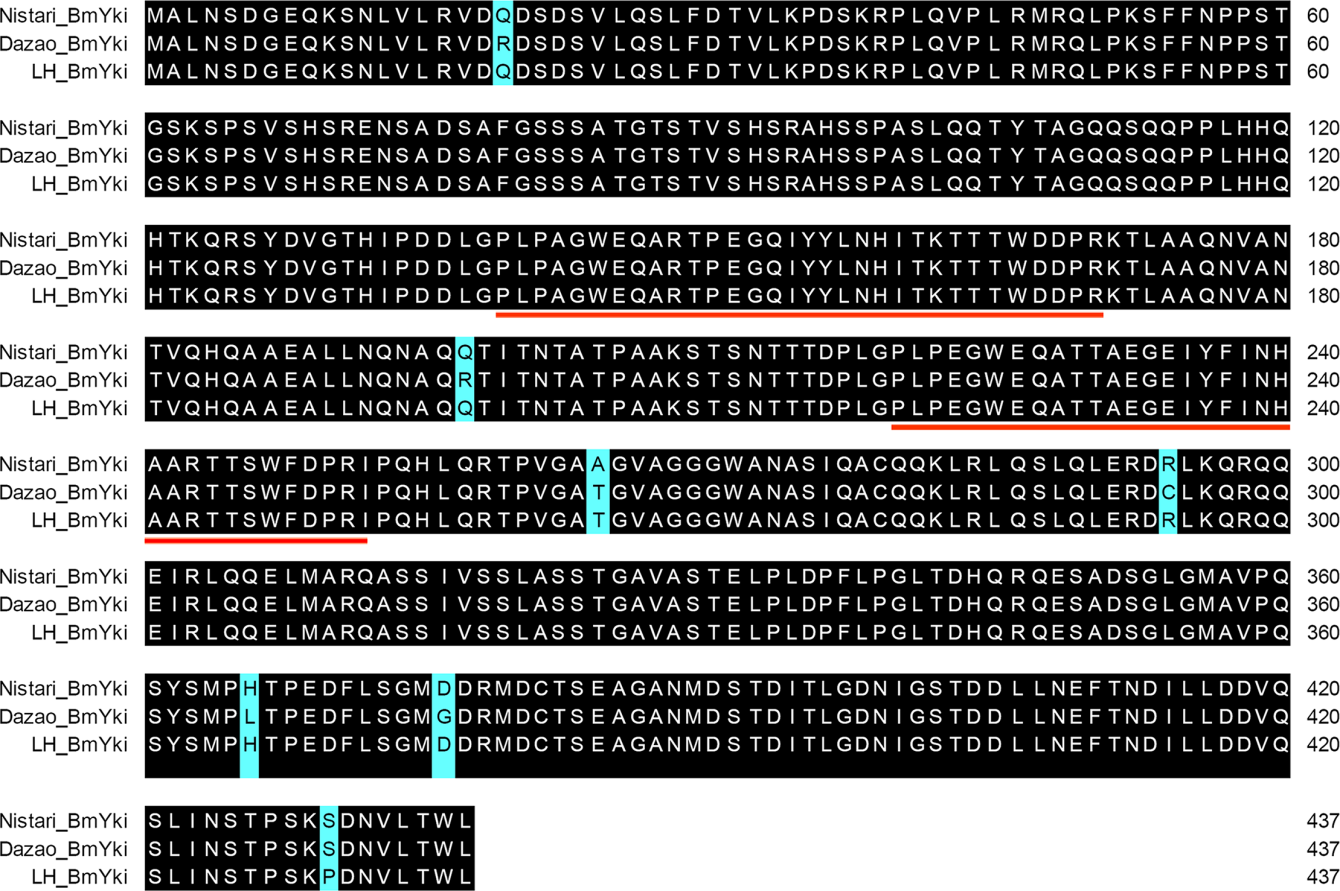
Alignment amino acid sequences of BmYki of three *B*. *mori* strains. Identical amino acid residues among *Dazao*, *Nistari* and *LH* strains are shaded in black. The WW domains are underlined in red.

To isolate the coding sequence of BmYki, the ~1300 bp fragment containing the putative *BmYki* gene was obtained by RT-PCR from three *B*. *mori* strains, respectively. Further sequence analysis showed that the coding sequence of each BmYki is 1314 bp in length, composed of six exons and five introns, and encodes a protein of 437 amino acids containing two conserved WW domains(Figs 1 and 2) that is capable of interacting with PPXY motifs found in Wts[21]. The amino acid sequence identity of the three *BmYki* genes was as high as 98.4%. It is worth noting that a ~1100 bp fragment was also amplified from each of the *B*. *mori* strain(S1 Fig), and nucleotide sequences of the fragment were almost identical to that of the *BmYki* gene, except for the lack of the third exon. We thus speculate that BmYki exists in multiple alternative splicing forms in *B*. *mori*, which is an interesting discovery that deserves further being investigated.

### Localization of BmYki-EGFP fusion proteins in BmN cells

**Fig 3:**
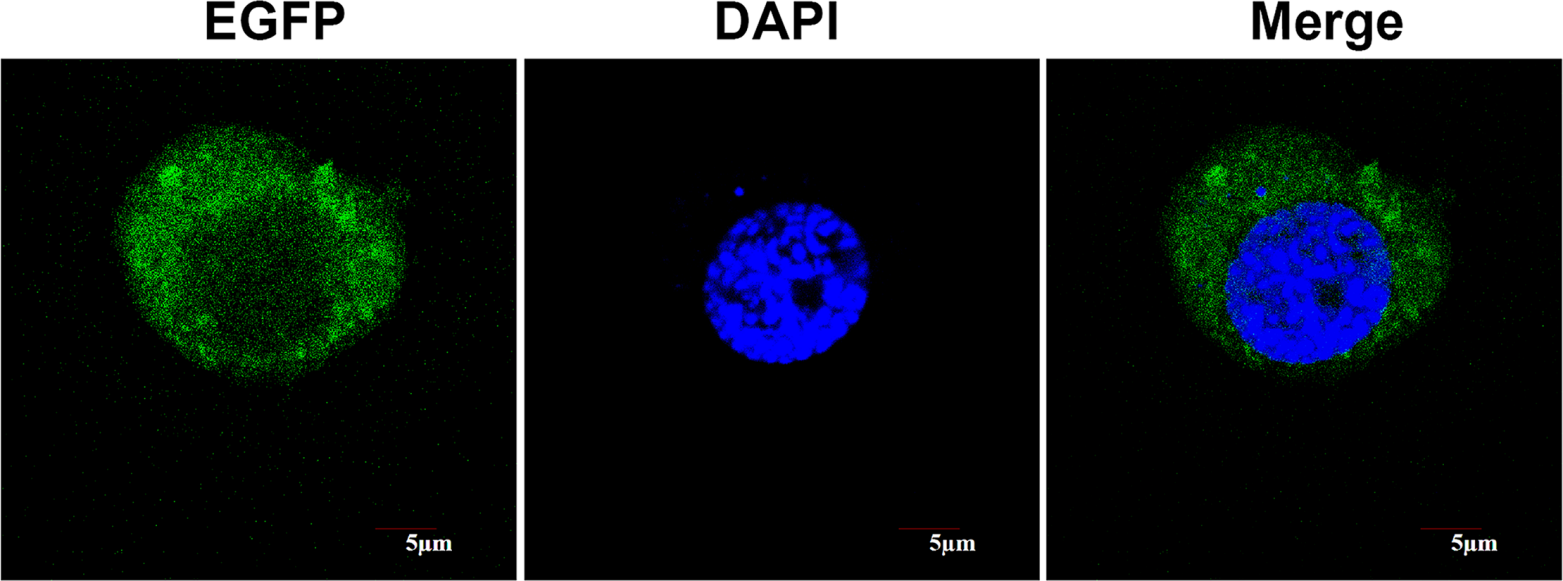
Localization of BmYki-EGFP fusion proteins in BmN cells. The coding sequence of the BmYki with the stop codon deleted was fused to EGFP and transiently transfected into the BmN cells. Subcelluar localization of the expressed BmYki-EGFP fusion proteins was observed by fluorescence microscopy.

It has been reported that Yki is mainly located in cytoplasm of *Drosophila* S2 cells[25]. To determine the subcellular localization of BmYki, the plasmid expressing BmYki-EGFP fusion proteins was transfected into BmN cells. The result showed that the BmYki-EGFP fusion protein was mainly cytoplasmic(Fig 3), which is similar to that observed in *Drosophila* S2 cells. The weak GFP fluorescence observed in the nucleus of BmN cells is thought to be the result of products working in the nucleus.

### Expression patterns of BmYki during development

**Fig 4:**
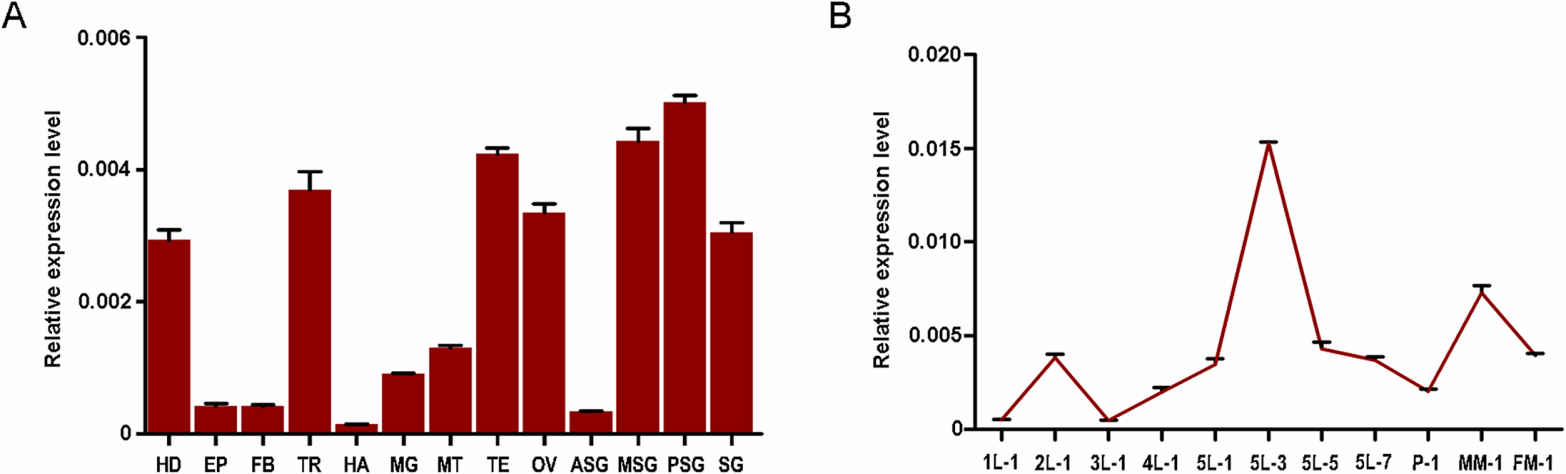
Expression profiles of *BmYki* in *B*. *mori*. (A)mRNA levels of *BmYki* in different tissues of *Dazao* strain. HD(head); EP(epidermis); FB (fat body); TR (trachea); HA(hemocyte); MG(midgut); MT(malpighian tubule); TE(testis); OV(ovary); ASG(anterior silk gland); MSG(middle silk gland); PSG(posterior silk gland); SG (total silk gland). (B)mRNA levels of *BmYki* at different developmental stages of *Dazao* strain. 1L-1, 2L-1, 3L-1, and 4L-1(day-1 of the first, second, third, and fourth larval instar); 5L-1, 5L-3, 5L-5, and 5L-7(day-1, 3, 5, and 7 of the fifth instar), P-1(day-1 of the pupal stage), MM-1 and FM-1(day-1 of the male and female moth). Relative mRNA levels of *BmYki* against *sw22934* are shown. Error bars represent mean ±SD of three samples.

To investigate the expression profile of BmYki, mRNA levels of *BmYki* gene in different tissues and various developmental stages of *B*. *mori* were determined by qRT-PCR. The results showed that *BmYki* transcripts were ubiquitous but less abundant in *B*. *mori*(Fig 4). Comparatively, *BmYki* was expressed higher in head, trachea, testis, ovary, and was particularly high in both the middle and posterior silk glands, and at the fifth-instar day-3 larvae—a developmental stage considered to be the beginning of mass synthesis of silk proteins, which strongly suggesting the involvement of BmYki in the regulation of silk protein synthesis. Further study is required to elucidate the meaning of BmYki in silk glands.

### Functional analysis of BmYki in BmE cells

**Fig 5:**
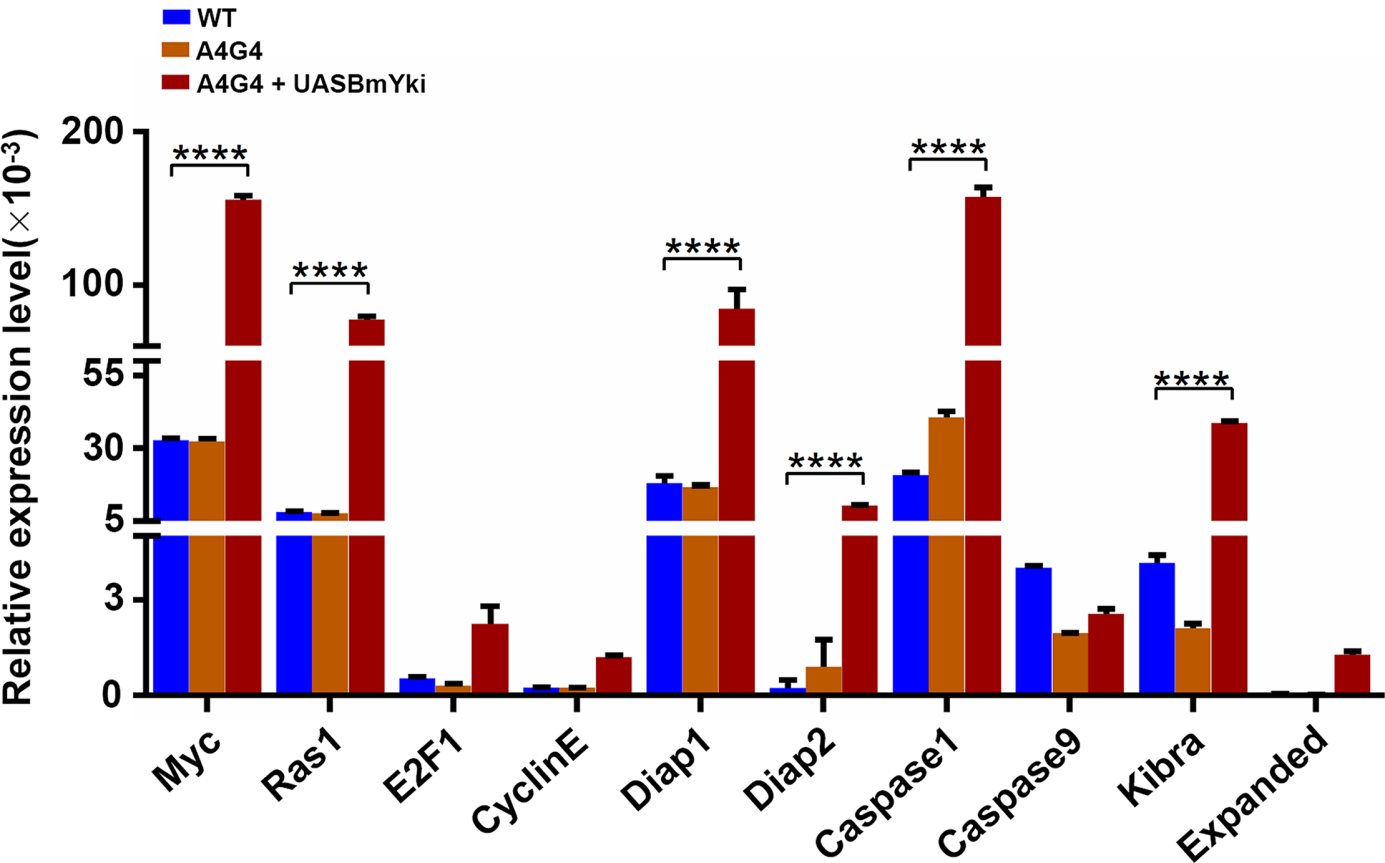
mRNA levels of Yki target genes in BmE cells overexpressing *BmYki*. Expression of *B*. *mori* genes homologous to known targets of *Drosophila* Yki. Relative mRNA levels are indicated as the ratios of mRNA levels between the target gene and *sw22934*. Error bars represent mean ±SD of three samples.

To investigate whether the BmYki is involved in the regulation of organ growth-related biological processes, mRNA levels of *B*. *mori* genes homologous to known targets of *Drosophila* Yki, including cell growth-promoting genes *Myc* and *Ras1*, cell cycle progression genes *E2F1* and *cyclinE*, cell apoptosis-related genes *Diap1*, *Diap2*, *Caspase1* and *Caspase9*, and Hpo pathway components *Expanded* and *Kibra*, were determined by qRT-PCR. As shown in Fig 5 and S2 Fig, nine of these ten genes were upregulated in BmE cells overexpressing *BmYki*, among which six targets *Myc*, *Ras1*, *Diap1*, *Diap2*, *Caspase1* and *Kibra* were remarkably upregulated, indicating that BmYki has the function of regulating downstream target genes associated with cell growth, proliferation, and apoptosis.

**Fig 6:**
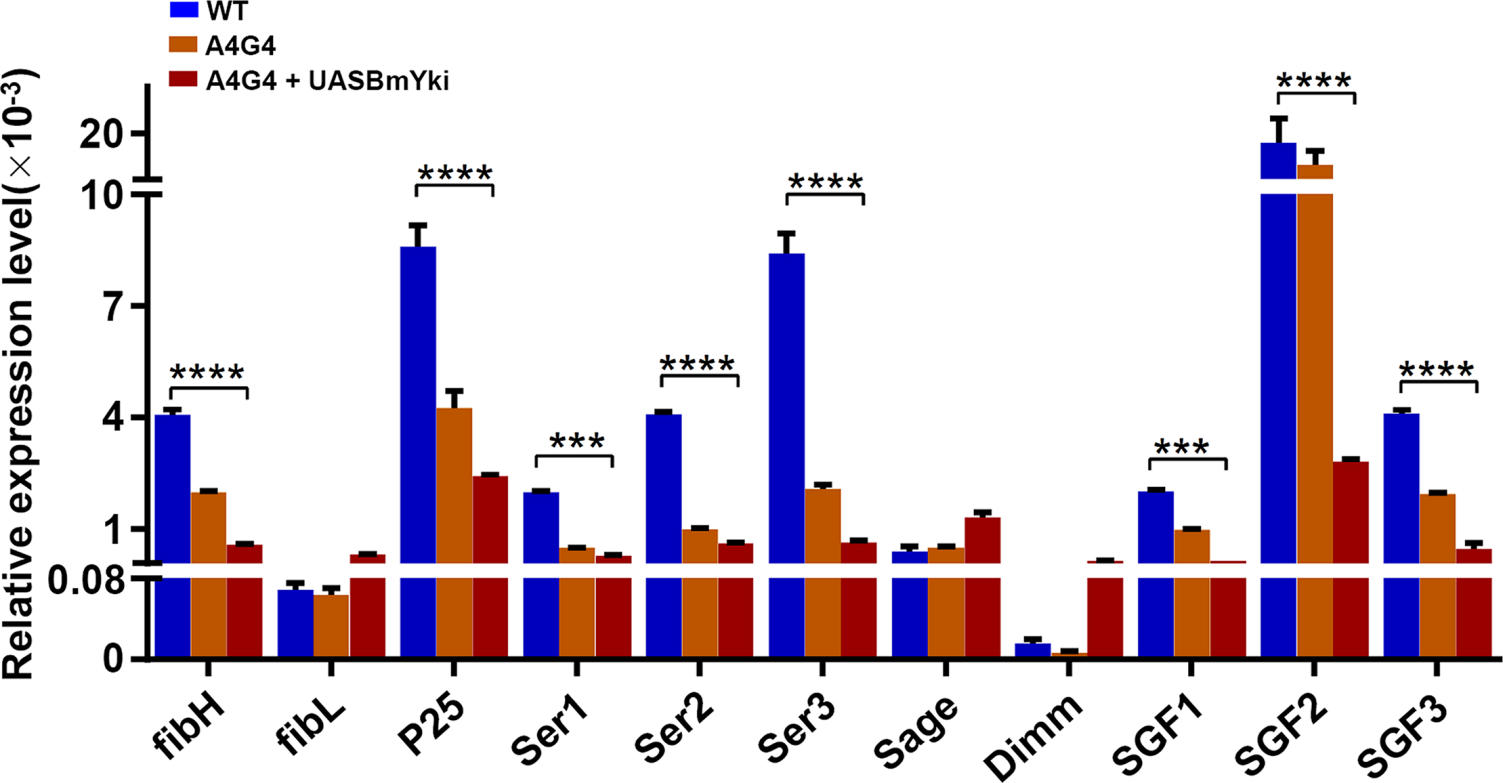
mRNA levels of silk protein synthesis-related genes in BmE cells overexpressing *BmYki*. Expression of silk protein synthesis-related genes in BmE cells were quantified by qRT-PCR. Relative mRNA levels are indicated as the ratios of mRNA levels between the target gene and *sw22934*. Error bars represent mean ±SD of three samples.

To further explore whether the BmYki is involved in silk protein synthesis, we examined the expression of genes responsible for synthesis of silk proteins, including fibroin protein-coding genes *FibH*, *FibL* and *P25*, sericin protein-coding genes *Ser1*, *Ser2* and *Ser3*, and silk gland factors *Sage*, *Dimm*, *SGF1*, *SGF2* and *SGF3*. The results showed that eight of eleven genes were significantly downregulated upon *BmYki* overexpression, while the other three genes were slightly upregulated(Fig 6), indicating that BmYki functions in the regulation of silk protein synthesis. We are now preparing to generate transgenic silkworms overexpressing *BmYki* specifically in the middle and posterior silk gland, to clarify the mechanism of BmYki in the regulation of silk protein synthesis.

## Conclusions

In conclusion, we isolated the coding sequence of BmYki with a length of 1314 bp from *B*. *mori*. Expression of the *BmYki* gene was comparatively high in both the middle and posterior silk gland, and at the fifth-instar day-3 larval stage. Furthermore, we showed that BmYki-EGFP fusion proteins were localized mainly in the cytoplasm of BmN cells. Finally, we demonstrated that overexpression of *BmYki* in BmE cells remarkably promoted the expression of genes homologous to known targets of *Drosophila* Yki, and significantly downregulated the expression of silk protein synthesis-related genes. This study provides new insights into the expression and function of BmYki. It would be very interesting to further elucidate the mechanism of BmYki in the regulation of silk protein synthesis.

## Supporting information

S1 Fig Agarose gel electrophoresis of the RT-PCR amplified *BmYki* gene products. Lane M: DNA marker; Lanes 1~2: cDNA template of *Dazao* embryos; Lanes 3~4: cDNA template of *LH* embryos; Lanes 5~10: cDNA template of *Nistari* middle silk glands; Lanes 11~14: cDNA template of *Nistari* posterior silk glands. RT-PCR amplification was performed at least three replicates for each sample. The PCR products were detected on 1.2% agarose gel electrophoresis. Red arrows indicate the ~1300 and ~1100 bp fragments containing the putative *BmYki* gene.
(TIF)

S2 Fig Detection of overexpression of *BmYki* in BmE cells. BmE cells transfected with UASBmYki and A4G4 plasmids were harvested to prepare cDNA templates. mRNA levels of *BmYki* were measured by qRT-PCR. Relative mRNA levels are indicated as the ratios of mRNA levels between the *BmYki* and *sw22934*. Error bars represent mean ±SD of three samples.
(TIF)
