# Supplementary figures and images for: Cloning and expression analysis of BmYki gene in silkworm, Bombyx mori
Source: PLoS One. 2017 Aug 9;12(8):e0182690. doi: 10.1371/journal.pone.0182690 (PMC5549978; doi:10.1371/journal.pone.0182690)

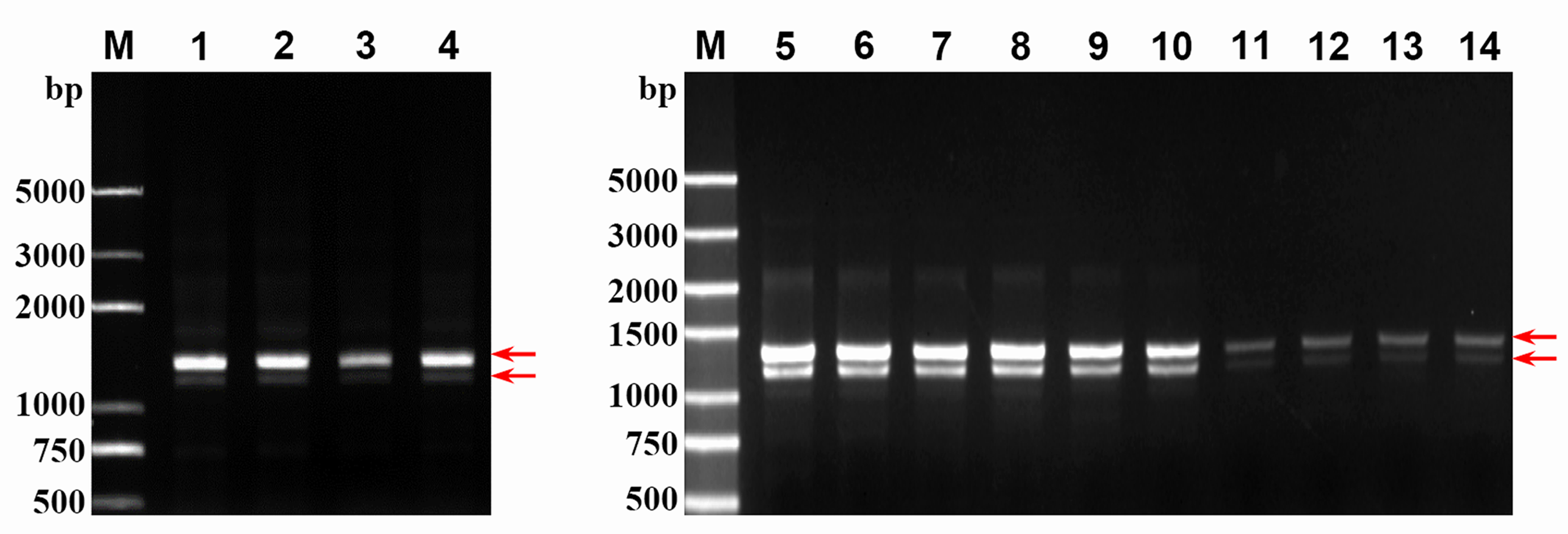

Supplement: S1 Fig — Lane M: DNA marker; Lanes 1~2: cDNA template of Dazao embryos; Lanes 3~4: cDNA template of LH embryos; Lanes 5~10: cDNA template of Nistari middle silk glands; Lanes 11~14: cDNA template of Nistari posterior silk glands. RT-PCR amplification was performed at least three replicates for each sample. The PCR products were detected on 1.2% agarose gel electrophoresis. Red arrows indicate the ~1300 and ~1100 bp fragments containing the putative BmYki gene. (TIF) [file pone.0182690.s001.tif]

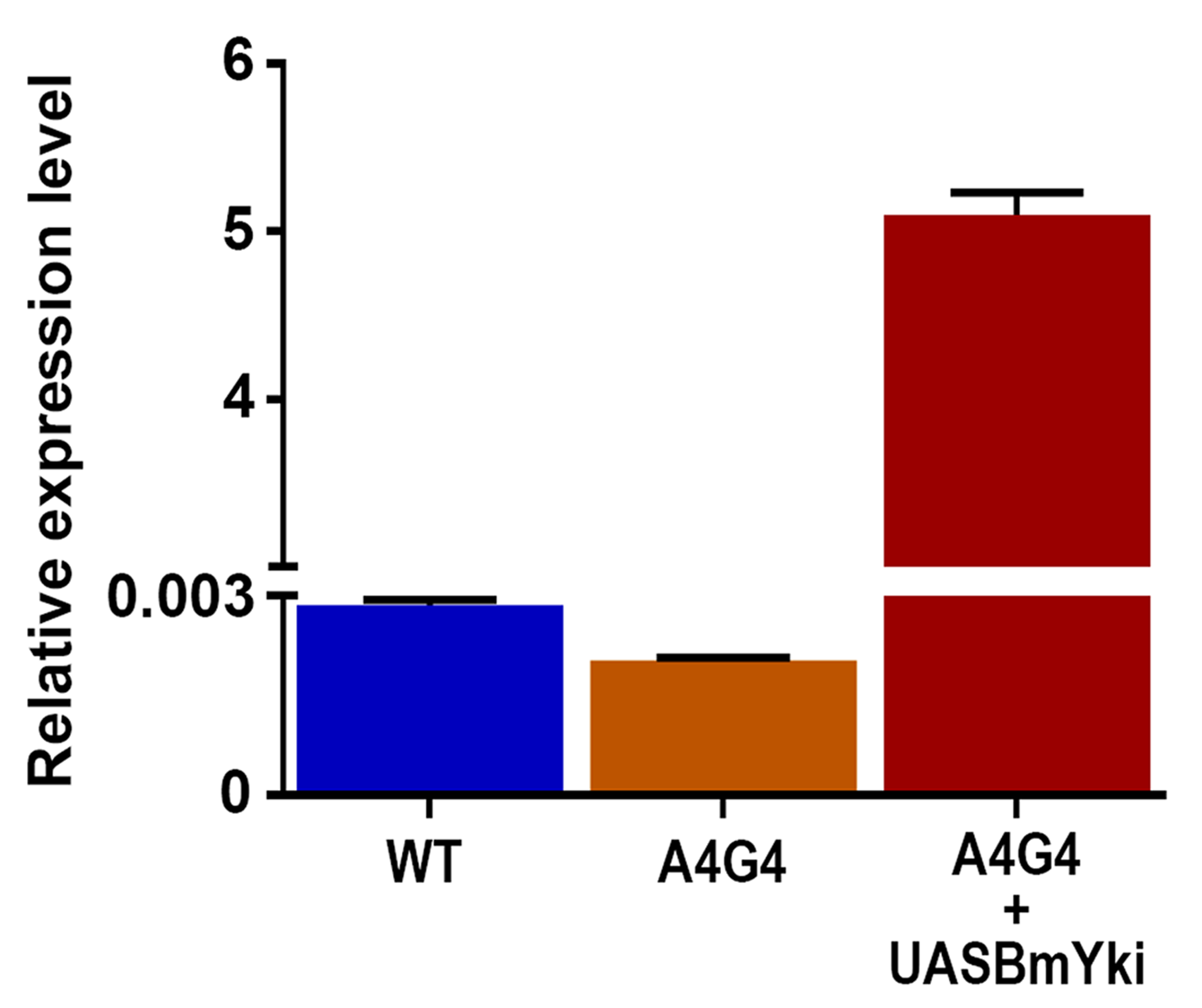

Supplement: S2 Fig — BmE cells transfected with UASBmYki and A4G4 plasmids were harvested to prepare cDNA templates. mRNA levels of BmYki were measured by qRT-PCR. Relative mRNA levels are indicated as the ratios of mRNA levels between the BmYki and sw22934. Error bars represent mean ±SD of three samples. (TIF) [file pone.0182690.s002.tif]
